# Morphological Ontogeny and Life Cycle of Laboratory-Maintained *Eremobelba eharai* (Acari: Oribatida: Eremobelbidae)

**DOI:** 10.3390/insects17010047

**Published:** 2025-12-30

**Authors:** Chang Chu, Yu Chen, Jun Chen

**Affiliations:** 1 State Key Laboratory of Animal Biodiversity Conservation and Integrated Pest Management, Institute of Zoology, Chinese Academy of Sciences, Beijing 100101, China; chuchang24@ioz.ac.cn (C.C.); chenyuwork_7@163.com (Y.C.); 2 College of Life Sciences, University of Chinese Academy of Sciences, Beijing 100049, China

**Keywords:** oribatid mite, morphology, juveniles, laboratory rearing, scanning electron microscopy

## Abstract

This study presents the first successful laboratory rearing of *Eremobelba eharai*, with the establishment of a sustainable multigenerational breeding system. We document for the first time its complete morphological ontogeny across all developmental stages (from larva to adult) and characterize its life cycle. We supplement the original adult description with detailed morphological characterization and illustrations of the gnathosomatic structures, including the subcapitulum, palps, and chelicerae. Scanning electron microscopy showed that its surface is covered with a granular cerotegument. Under isolated rearing conditions, this species can complete the entire egg-to-egg developmental cycle. In addition, preliminary behavioral observations during rearing revealed preferences for dark environments, characteristic leg-shaking movements, and gregarious oviposition on active dry yeast particles, with no evidence of cannibalism.

## Simple Summary

The oribatid mite, *Eremobelba eharai*, is distributed in northern China. Previously, information on its laboratory rearing and reproductive biology was lacking. Based on the first successful multigenerational laboratory rearing of this species, we thoroughly documented the morphological changes across all developmental stages, from larva to adult, and characterized its complete life cycle.

## 1. Introduction

The study of oribatid mite ontogeny can be traced back to the 19th century, when C. L. Koch [1,2,3,4,5,6] first documented the morphological characteristics of oribatid juveniles in his work German Crustaceans, Myriapods, and Arachnids. However, early researchers often did not recognize the connection between juvenile and adult stages, leading to the establishment of numerous synonyms and *species inquirendae*; for instance, the tritonymph and adult of *Camisia (Camisia) biurus biurus* (Koch, 1839) exhibit significant morphological differences. This disparity directly led to the misidentification of its tritonymph as *Nothrus furcatus* Koch, 1839, a taxonomic error that resulted in the creation of a synonym [3,7]. In 1953, Grandjean established a classification system for oribatid mites based on morphological and developmental characteristics, which significantly advanced the understanding of taxonomic methodology for this group [8]. Grandjean also discussed the taxonomic value and evolutionary implications of certain key morphological traits [8]. Similarly, Travé [9] demonstrated through a series of examples that complete ontogenetic data are essential for constructing reliable classification systems and for accurately understanding the ecological niches and life history strategies of species.

It is noteworthy that comprehensive studies on oribatid mite ontogeny are still limited. According to a 2024 review, only 1072 species of oribatid mites have been examined for morphological descriptions of juvenile stages [10]. Additionally, only 144 species have been investigated with respect to their life cycles and lifespans, as documented in a 2021 review [11]. Both numbers are extremely low compared to the more than 10,000 known species of oribatid mites [12]. This limitation can be attributed in part to the lack of sufficient taxonomic expertise in identifying different developmental instars, which has hindered progress in the field. Moreover, due to their low fecundity, complex and poorly defined feeding habits, and long lifespan, together with the fact that only a few species have been successfully reared under laboratory conditions to obtain reliable life history data, oribatid mites are difficult to rear under artificial conditions [11,13,14,15,16].

Within the family Eremobelbidae, research on juvenile morphology remains extremely uncommon. While morphological ontogeny has been documented for several species, existing studies contain significant gaps. For *Eremobelba geographica* Berlese, 1908, juvenile ecology and biology were investigated by Bulanova-Zachvatkina and Shereef [17] and Shereef [18]; subsequently, Weigmann [19] described the nymphs and illustrated the tritonymph. Hammer [20] described and illustrated the protonymph of *E. foliata* Hammer, 1958. Ecological and biological observations of *E. gracilior* Berlese, 1908 were recorded by Hartenstein [21]. Notably, these studies did not cover all developmental stages for their respective species, and available descriptions and illustrations of documented instars remain too general to permit detailed morphological comparisons. A recent breakthrough came from Seniczak et al. [22], who conducted the first comprehensive study of *E. geographica*, documenting its complete morphological ontogeny and comparing adult morphology with related species. To date, only two species, *E. geographica* and *E. gracilior*, have had their full life cycles examined [18,21], with development from egg to adult requiring 56–74 days and 68–75 days, respectively.

Chen and Gao described *Eremobelba eharai* as a new species in 2017 based on adult specimens from Liaoning, northeastern China. However, this original description was limited to adults and remained relatively general; for instance, details of the chelicerae and palps were not provided. Additionally, its juvenile morphology and complete life cycle retain entirely unknown. In the present study, we aim to supplement this description and its illustration and to document the ontogeny of the species based on specimens from our laboratory culture. We provide scanning electron microscopy (SEM) images and present observations on its development and behavior.

## 2. Materials and Methods

### 2.1. Collection and Identification of Eremobelba eharai

A soil sample was collected under a pine tree (40°0′8.15″ N, 116°25′50.29″ E, 41.20 m) in Huangcaowan Countryside Park, Chaoyang District, Beijing, China, on 10 August 2021. Living mites were extracted using a Berlese–Tullgren funnel (IZCAS, Beijing, China) and placed in 250 mL plastic bottles with wet cotton at the bottom. A preliminary identification was conducted under a Leica EZ4 stereomicroscope (Leica Microsystems, Wetzlar, Germany), and ca. 300 adult individuals were judged to belong to the same species. To confirm the species identity, 50 adults were randomly selected from this stock population, mounted in lactic acid, and examined under a Leica DM2500 compound microscope (Leica Microsystems, Wetzlar, Germany) using standard taxonomic procedures. All examined specimens were confirmed as *Eremobelba eharai* Chen & Gao, 2017. Specimens used for morphological measurement and description (*n* = 10) were preserved in 75% ethanol and deposited in the Institute of Zoology, Chinese Academy of Sciences (IZCAS), Beijing, China.

### 2.2. Morphology of Eremobelba eharai

#### 2.2.1. Observation, Documentation, and Terminology

For morphological examination, all instars were cleared and mounted in lactic acid on temporary cavity slides. Observations, measurements, and illustrations were performed using a Leica DM2500 compound microscope. All measurements are given in micrometers (µm). We measured the following morphological traits: body length and prodorsum length in lateral view, from the rostrum tip to the posterior margin of the notogaster (or gastronotum in juveniles) and to the posterior margin of the gastronotum, respectively; notogastral (gastronotal in juveniles) width and body width in dorsal view, both representing the maximum distance between the anterior and posterior margins; and genital opening length and anal opening length in ventral view. Additionally, when measuring setae, their curvature was compensated for by taking the perpendicular distance relative to their longitudinal axis. To ensure accuracy, specimens were optimally positioned to eliminate parallax error during the examination and measurement of other structures. The Mean and Standard Deviation (SD) of some morphological characters of juvenile stages and adult of *Eremobelba eharai* were calculated. Formulas for leg setation are given in parentheses according to the sequence trochanter–femur–genu–tibia–tarsus (famulus included); formulas for leg solenidia are given in square brackets according to the sequence genu–tibia–tarsus. We restricted the accompanying illustrations to body regions of *E. eharai* instars that show marked ontogenetic variation. These include dorsal and lateral aspects, as well as some leg segments, of the larva, tritonymph, and adult; the ventral morphology of all instars; and the adult palp and chelicera.

Morphological terminology applied herein adheres to the frameworks established in recent works on the genus *Eremobelba* [23,24], with general concepts following those of Norton and Behan-Pelletier [16] and leg chaetotaxy following Norton [25].

#### 2.2.2. Scanning Electron Microscopy (SEM) Sample Preparation

We provide SEM images of this species covering all life stages, as well as detailed SEM images of specific structures of the adult. The specimens used for SEM were collected from the rearing containers, cleaned with a fine brush, and then immersed in 70%, 80%, and 90% ethanol for 1 h each, respectively, and finally in fresh 100% ethanol for 24 h. The samples were then subjected to critical point drying using an Automated Critical Point Dryer (Leica EM CPD300, Leica Microsystems, Wetzlar, Germany) and mounted onto aluminum sample stubs with double-sided conductive carbon tape. A gold–palladium mixture coating was applied to the surface using a Super Cool Sputter Coater (Leica EM SCD050, Leica Microsystems, Wetzlar, Germany). Microscopic images were captured with a Scanning Electron Microscope (FEI Quanta 450, Thermo Fisher Scientific, Hillsboro, OR, USA).

### 2.3. Developmental Biology and Behavior of Eremobelba eharai

#### 2.3.1. Living Mites Cultivation

These adult mites were kept in multiple 250 mL plastic bottles with a mixture of gypsum powder and activated carbon (9:1) at the bottom [26]. Small holes were made in the bottle caps with a needle to ensure adequate air exchange. The rearing conditions were set to a temperature of 25 ± 3 °C, a relative humidity of 80 ± 5%, and a dark environment. *E. eharai* was fed with active dry yeast once a week, and any leftover food was removed to prevent the growth of mold.

#### 2.3.2. Life Cycle Experiment and Developmental Monitoring

In the life cycle experiment, 200 adult individuals were randomly selected from the laboratory population of *E. eharai* and transferred to a new rearing container. To ensure an adequate sample size and facilitate accurate developmental recording, the following procedure was adopted: the container was inspected daily for oviposition, and if fewer than 50 eggs were produced, all eggs were discarded; if more than 50 eggs were laid, they were transferred to a new container for hatching. A total of 60 eggs and 70 eggs were obtained on 16 July 2024 and 19 July 2024, respectively. Eggs were examined for hatching every 24 h. Newly hatched larvae were individually reared until the resulting adults produced offspring, and the transition dates between successive developmental stages were recorded. After completion of the life cycle experiment, all individuals used in the rearing trial (*n* = 91 adult mites) were re-examined using the same lactic acid mounting and compound microscope method, and all were reconfirmed as *E. eharai*, ensuring that no other species were present during the rearing experiment.

The experiment was conducted for only one generation and continued from egg hatching until all individuals of the offspring generation had reached adulthood. No additional generations were reared.

#### 2.3.3. Statistical Analysis

Individuals that died during development were excluded from the analysis, and only those that successfully reached adulthood were used to calculate the life cycle durations. The duration of each life stage was calculated using the following formulae:

Stage Duration (Egg Duration, Larva Duration, Protonymph Duration, Deutonymph Duration, Tritonymph Duration) = Start date of the current stage to the start date of the next stage.

Quiescent Duration (Larva Quiescent, Protonymph Quiescent, Deutonymph Quiescent, Tritonymph Quiescent) = Start date of the quiescent phase of the current stage to the start date of the next stage.

Total Immature Duration = Start date of the egg stage to the start date of the adult stage.

Adult–Egg = Start date of the adult stage to the first oviposition date.

Egg–Egg = Start date of the egg stage to the oviposition date.

All data were processed and analyzed using Python 3.11.8 (Python Software Foundation, Wilmington, DE, USA). The Mean and Standard Error (SE) of each life stage were calculated. The 95% confidence intervals (CIs) were calculated based on the SE, assuming a normal distribution of the data (*n* = 91).

#### 2.3.4. Preliminary Behavioral Observations

Supplementary behavioral observations were conducted opportunistically during the routine maintenance and monitoring of the *E. eharai* cultivation. These observations were opportunistic, non-quantitative, and aimed at providing preliminary insights into the species’ ethology. The phototactic response was assessed by examining mite distribution within rearing containers using a stereomicroscope and recording their movement reactions upon exposure to light during handling. Oviposition behavior was examined by recording the location and spatial patterning of eggs during daily egg checks. Distinct locomotory patterns and the presence or absence of cannibalism were noted during microscopic examinations of individuals and during general rearing observations under both food-present and food-absent conditions.

## 3. Results

Family Eremobelbidae Balogh, 1961

Genus *Eremobelba* Berlese, 1908

Type species: *Eremaeus leporosus* Haller, 1884

*Eremobelba eharai* Chen & Gao, 2017

### 3.1. Morphology and Ontogeny of Eremobelba eharai

#### 3.1.1. Supplementary Diagnosis

In the adult, prodorsum with a pair of exobothridial setae (*ex*), epimeral setal formula 3–1–3–3, seta *1c* present; lateral tubercles (*lt*) and anterior ventrosejugal tubercles (*Va*), middle ventrosejugal tubercles (*Vm*) and posterior ventrosejugal tubercles (*Vp*) present, with *Vm* and *Vp* bearing epimeral setae *3c* and *4c*, respectively. Aggenitoadanal region with 17–19 pairs of neotrichous setae, among them, eight pairs of setae with three to six stellate branches.

In the juvenile, prodorsal seta *ex* smooth, bothridial seta (*bs*) barbed bilaterally. In the larva, gastronotal seta *c*_3_ long, with a pair of anal valve setae; all setae inserted on small apophyses, except for seta *dp* inserted on large apophysis. In the nymphs, gastronotal seta *c*_3_ vestigial, with only alveolus remaining visible; all setae of protonymph, and gastronotal setae of deutonymph and tritonymph inserted on small apophyses.

In addition to the distinctive characters mentioned by Chen and Gao [27], the species can also be distinguished from *E. japonica* Aoki, 1959 by the setae *le* inserted on a pair of long, inverted L-shaped ridges (vs. the setae *le* inserted on a pair of small, circular ridges in *E. japonica*). This feature is consistent with the illustration provided in Chen and Gao [27].

#### 3.1.2. Redescription of Adult

Measurements. Body length 632 (590–670; *n* = 10); notogastral width 363 (340–382; *n* = 10).

**Figure 1:**
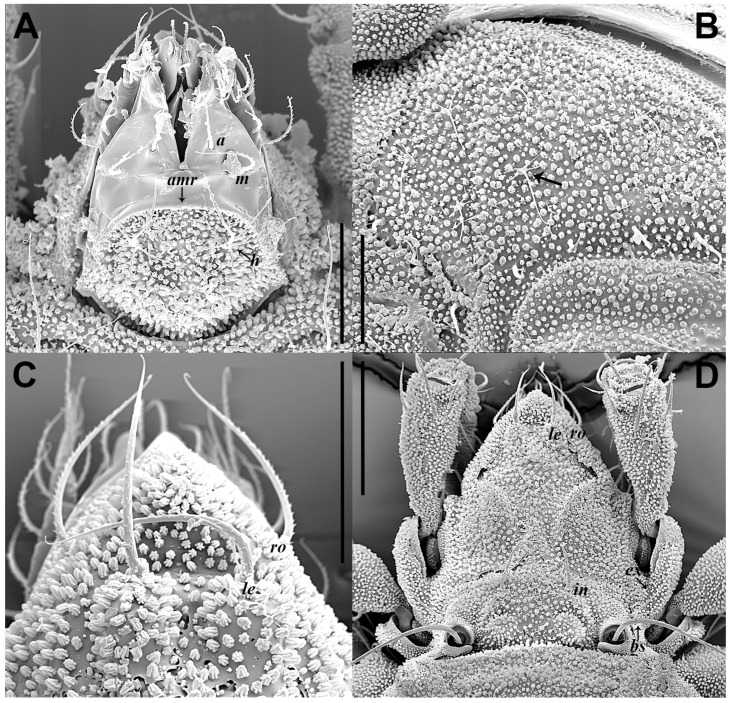
*Eremobelba eharai*, adult, Scanning Electron Microscopy (SEM) micrographs: (**A**) gnathosoma, ventral view; (**B**) part of aggenitoadanal region, ventral view, arrow points at neotrichous seta; (**C**) rostrum region, dorsal view; (**D**) prodorsum, dorsal view. Scale bars: (**A**–**C**) = 50 µm; (**D**) = 100 µm.

**Figure 2:**
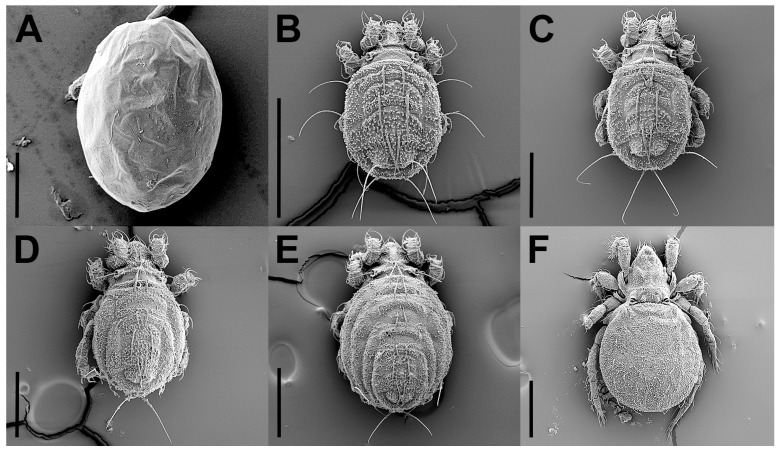
*Eremobelba eharai*, life cycle, SEM micrographs: (**A**) egg; (**B**) larva, dorsal view; (**C**) protonymph, dorsal view; (**D**) deutonymph, dorsal view; (**E**) tritonymph, dorsal view; (**F**) adult, dorsal view. Scale bars: (**A**) = 50 µm; (**B**–**F**) = 200 µm.

Integument (Figure 1 and Figure 2F). Body color yellowish brown. Body and legs covered by densely granular cerotegument.

**Figure 3:**
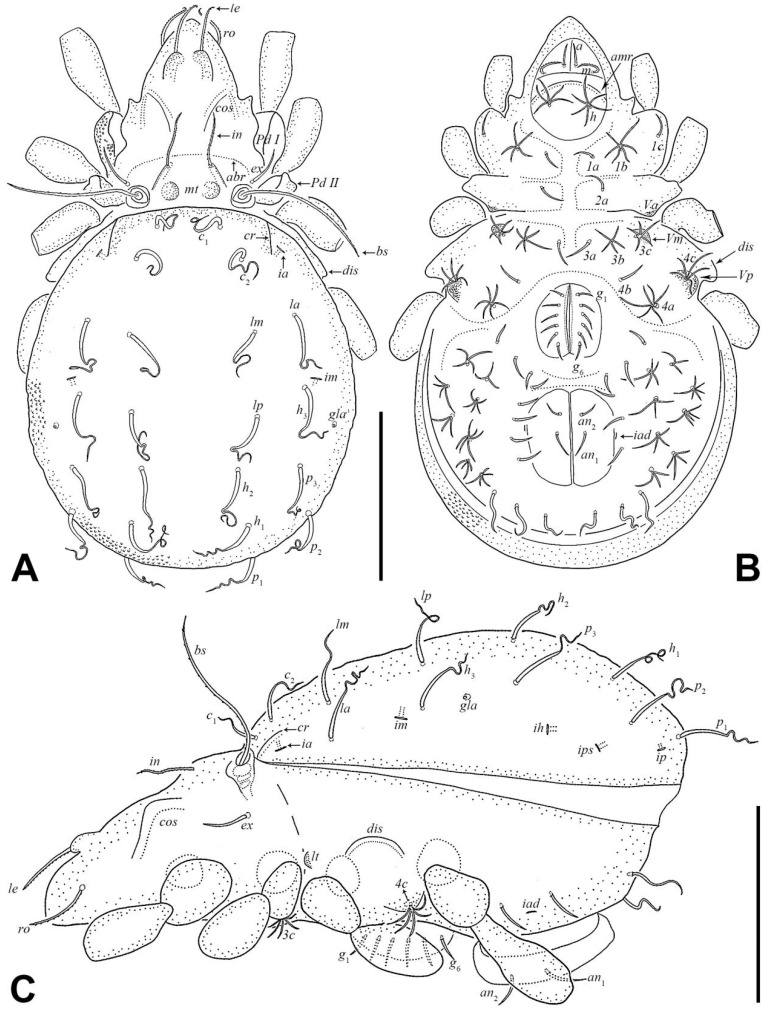
*Eremobelba eharai*, adult, scale bars 100 µm: (**A**) dorsal view (legs partially omitted); (**B**) ventral view (legs and gnathosoma partially omitted); (**C**) lateral view (legs partially omitted).

Prodorsum (Figure 1C,D and Figure 3A). Rostral seta (*ro*) and lamellar seta (*le*) setiform, slightly barbed unilaterally, *ro* curved medially, and *le* inserted on a pair of separated ridges which connected without transverse ridge; interlamellar seta (*in*) setiform, slightly barbed bilaterally. Costula (*cos*) situated between setae *le* and *in*. Bothridial seta (*bs*) flagellate, slightly barbed unilaterally, longer than distance between bothridia. Exobothridial seta (*ex*) setiform, smooth. A pair of mediobasal tubercles (*mt*) arising between bothridia, and another pair of anterobothridial ridges (*abr*) originated from lateral–anterior margin of bothridia and extending to central part of prodorsum anterior to *in*.

Notogaster (Figure 3A). Dorsosejugal furrow slightly convex, with a pair of slightly developed humeral cristae (*cr*). Eleven pairs of notogastral setae flagellate, with slender tips curved one to three times. Setae *c*_1_ and *c*_2_ shorter than other notogastral setae. Lyrifissures and opisthonotal gland openings (*gla*) visible, *ia* located laterally to *cr*; *im* between setae *la* and *h*_3_; *ih* and *ips* close to each other and lateral to seta *p*_2_; *ip* between setae *p*_1_ and *p*_2_; *gla* between setae *h*_3_ and *p*_3_.

**Figure 4:**
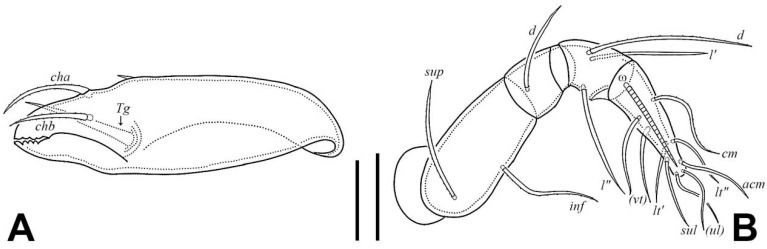
*Eremobelba eharai*, adult, scale bars 10 µm: (**A**) chelicera, left, antiaxial view; (**B**) palp, right, antiaxial view.

Gnathosoma (Figure 1A, Figure 3B, and Figure 4). Subcapitular seta *a* setiform, smooth; *m* setiform and slightly barbed unilaterally, strongly curved medially; *h* stellate with branches. Anterior region of mentum with ridge (*amr*). Chelicerae chelate, *cha* slightly longer than *chb*, both barbed bilaterally. Palp relatively small and thin, setae *sup* and *inf* on femur, *d* on tibia and *cm* on tarsus barbed unilaterally, other setae smooth. Palps with setation 0–2–1–3–9(+ω).

Epimeral and lateral podosomal regions (Figure 3B,C). One pair of *lt* present at sejugal suture; discidium (*dis*) located between legs III and IV, lateral to *Vp*. Ventrosejugal tubercles *Va*, *Vm*, and *Vp* present, with *Vm* and *Vp* bearing epimeral setae *3c* and *4c*, respectively. Epimeral setal formula 3–1–3–3. Epimeral setae *1a*, *1c*, *2a*, *3a*, and *4b* setiform; *1b*, *3b*, *3c*, *4a*, and *4c* stellate with five to six branches.

Anogenital region (Figure 1B and Figure 3B). Six pairs of genital setae (*g*) and two pairs of anal setae (*an*) setiform, smooth. Aggenitoadanal region with 17–19 pairs of neotrichous setae, among them: three pairs of setae near to genital aperture; three to five pairs of setae near to anal aperture, and number of setae near to anal aperture with variation among specimens: left with five setae and right with three setae; three pairs of setae flagellate near to posterior edge of ventral plate; eight pairs of setae with three to six stellate branches at middle region of ventral plate. Adanal lyrifissure (*iad*) para-anal and close to middle region of anal aperture.

**Figure 5:**
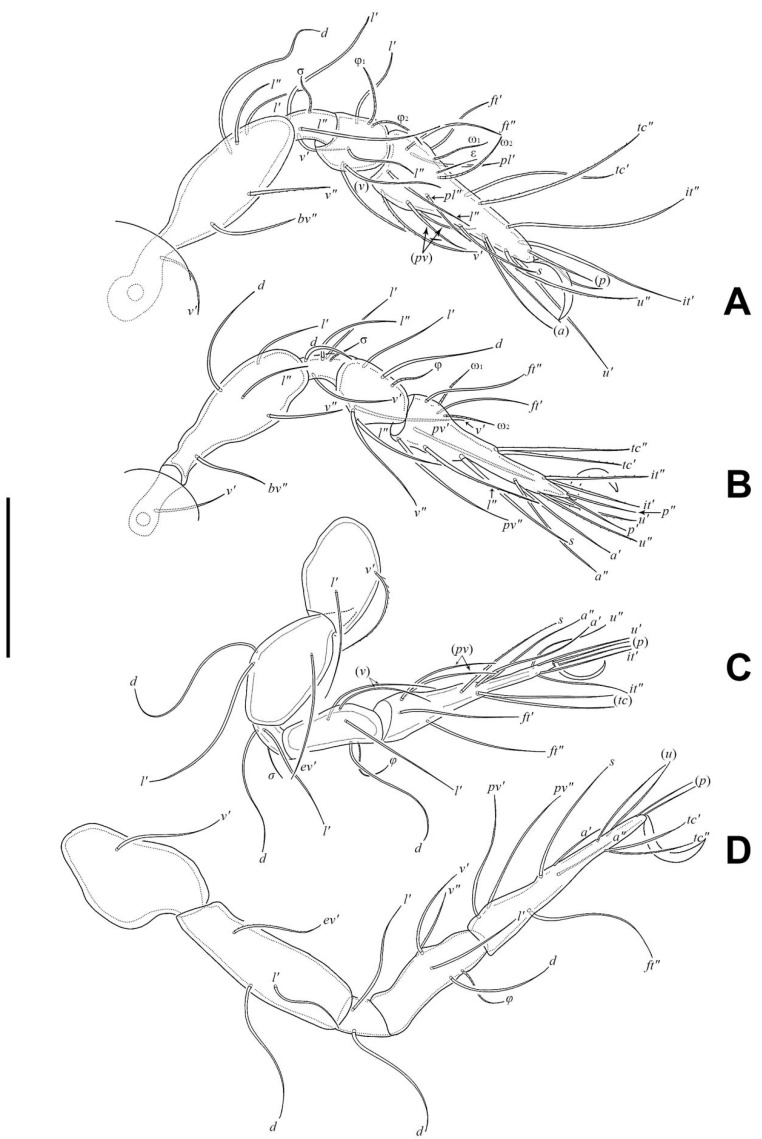
*Eremobelba eharai*, adult, right, antiaxial view, scale bar 50 µm: (**A**) Leg I; (**B**) Leg II; (**C**) Leg III; (**D**) Leg IV.

Legs (Figure 5). Formulae of leg setation and solenidia: I (1–5–3–4–20) [1,2], II (1–5–4–5–16) [1,2], III (2–3–2–4–15) [0,1], IV (1–3–2–4–12) [0–1–0]. Legs monodactylous. Seta *d* present on all genua and tibiae, except for leg I.

Remarks. Adult similar to that investigated by Chen and Gao [27], but in our specimens, prodorsal setae *ro*, *le* and *bs* slightly barbed unilaterally, *in* slightly barbed bilaterally (vs. smooth in the original description), prodorsal seta *ex* present (vs. absent); gnathosoma with *amr* (vs. absent); epimeral setal formula 3–1–3–3 (vs. 2–1–3–3); sejugal suture with a pair of *lt*, *Va*, *Vm*, and *Vp* (vs. absent); aggenitoadanal region with 17–19 pairs of neotrichous setae (vs. 16–17).

#### 3.1.3. Description of Juveniles

**Figure 6:**
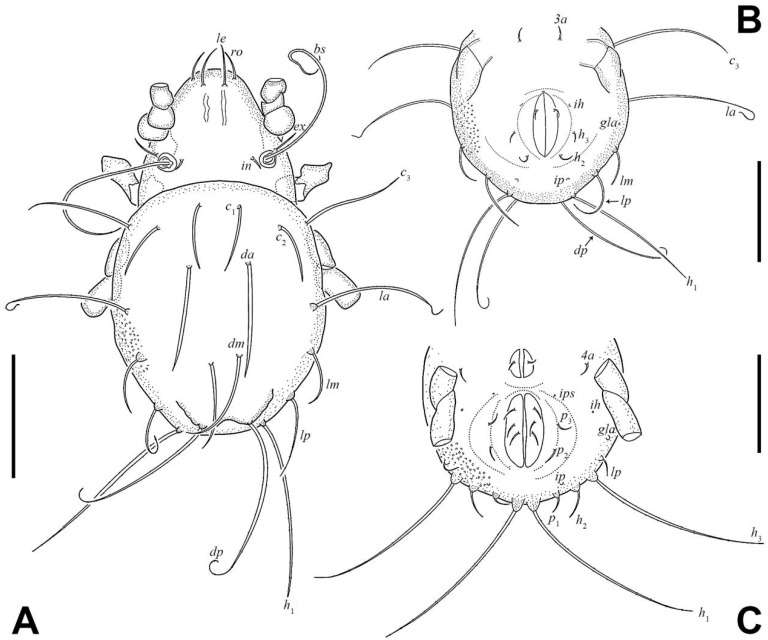
*Eremobelba eharai*, scale bars 50 µm: (**A**) larva, dorsal view (legs partially omitted); (**B**) larva, ventral view (front half omitted; legs partially omitted); (**C**) protonymph, ventral view (front half omitted; legs partially omitted).

**Figure 7:**
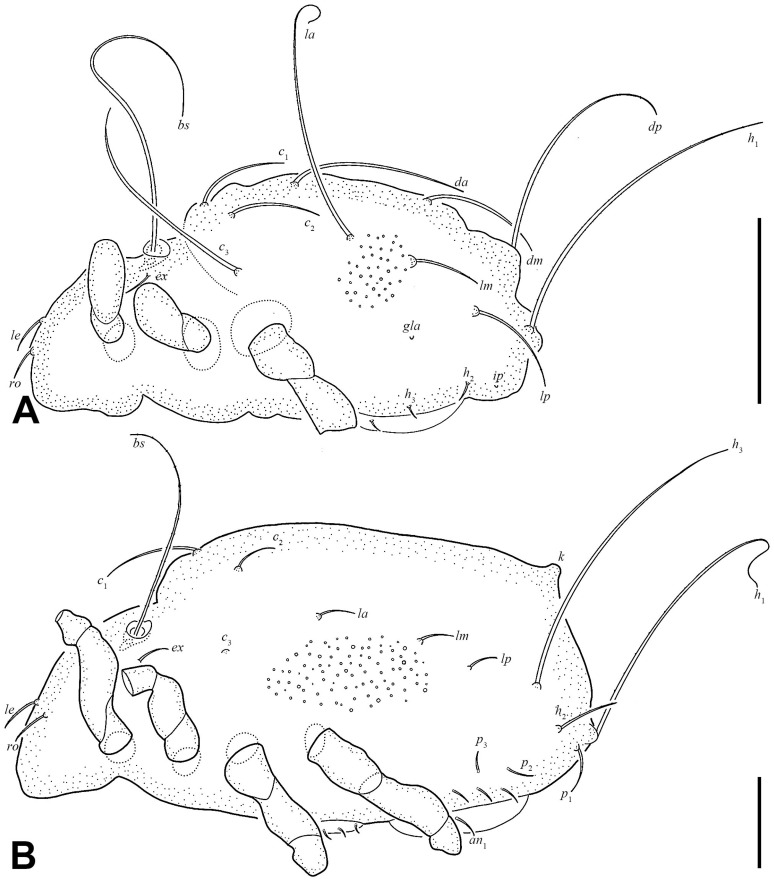
*Eremobelba eharai*, scale bars 50 µm: (**A**) larva, lateral view (legs partially omitted); (**B**) tritonymph, lateral view (legs partially omitted).

**Figure 8:**
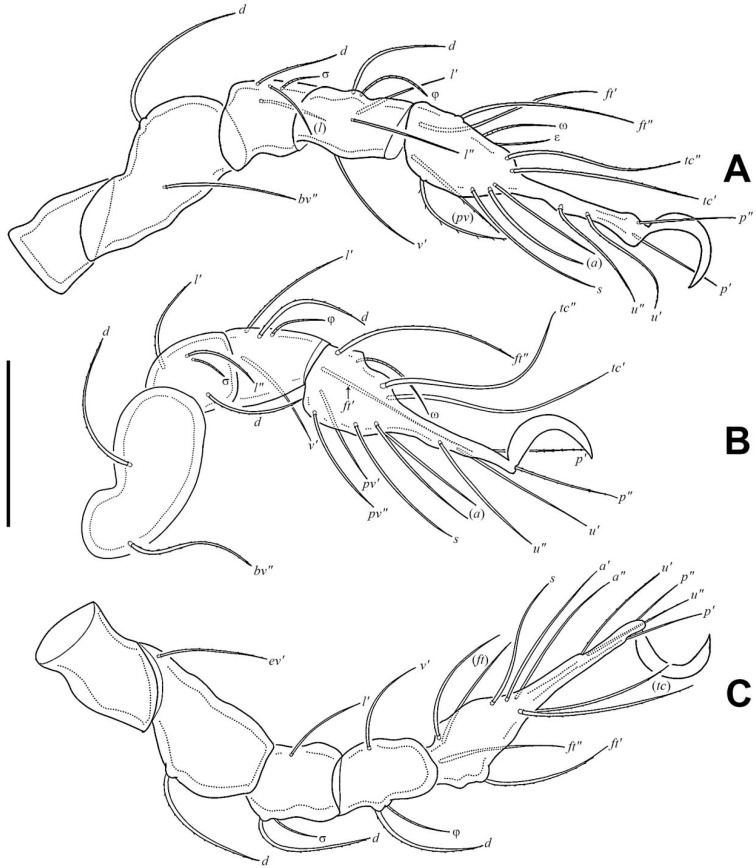
*Eremobelba eharai*, larva, right, antiaxial view, scale bar 20 µm: (**A**) Leg I; (**B**) Leg II; (**C**) Leg III.

Larva (Figure 2B, Figure 6A,B, Figure 7A, and Figure 8; Table 1). Oval in dorsal aspect and unpigmented. Prodorsum subtriangular, prodorsal setae *ro*, *le*, *in*, and *ex* smooth, *in* short, *ro*, *le*, and *ex* middle. Mutual distance between setae *ro* about two times longer than that between setae *le*. Bothridium oval, seta *bs* flagellate and slightly barbed bilaterally. Gastronotum with 12 pairs of setae, including *h*_2_ and *h*_3_ inserted lateral to anal valves. Setae *h*_2_ and *h*_3_ short, *c*_1_, *c*_2_, *lm*, *lp*, and *dm* of middle, other setae long; *c*_1_, *c*_2_, *da*, *dm*, *h*_2,_ and *h*_3_ smooth, *c*_3_, *la*, and *lm* slightly barbed unilaterally, other setae barbed bilaterally. All setae inserted on small apophyses, except for seta *dp* inserted on large apophysis. Anal valves with a pair of setae. Cupules *ia* and *im* not observed in granular cerotegument, cupule *ih* lateral–anterior part of anal valves, cupule *ip* between setae *h*_1_ and *h*_2_, *gla* lateroventral to region between setae *la* and *lm*. Formulae of leg setation and solenidia: I (0–2–3–4–14) [1], II (0–2–3–3–12) [1], III (0–2–2–2–13) [0,1]. Legs monodactylous. Seta *d* present on all genua and tibiae.

**Figure 9:**
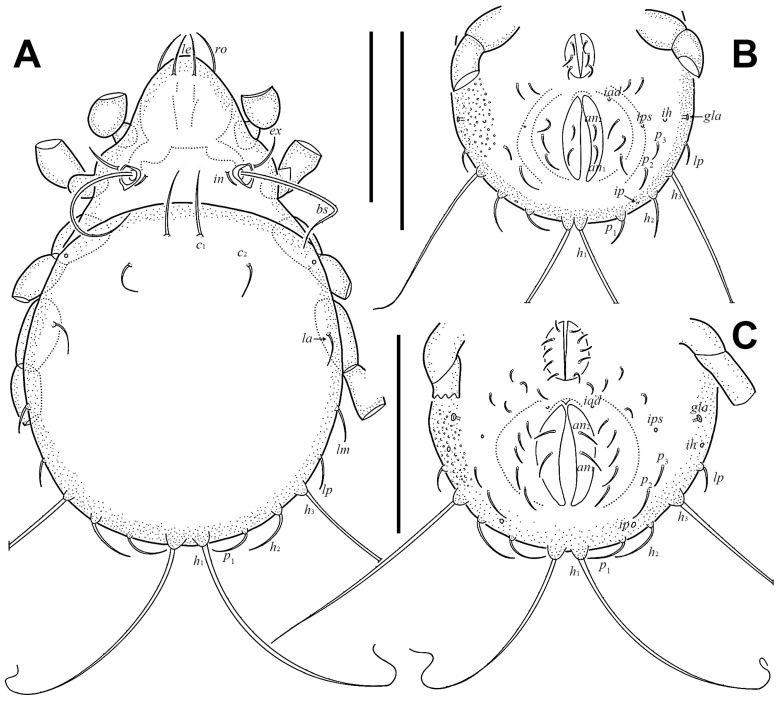
*Eremobelba eharai*, scale bars 50 µm: (**A**) tritonymph, dorsal view (legs partially omitted); (**B**) deutonymph, ventral view (front half omitted; legs partially omitted); (**C**) tritonymph, ventral view (front half omitted; legs partially omitted).

**Figure 10:**
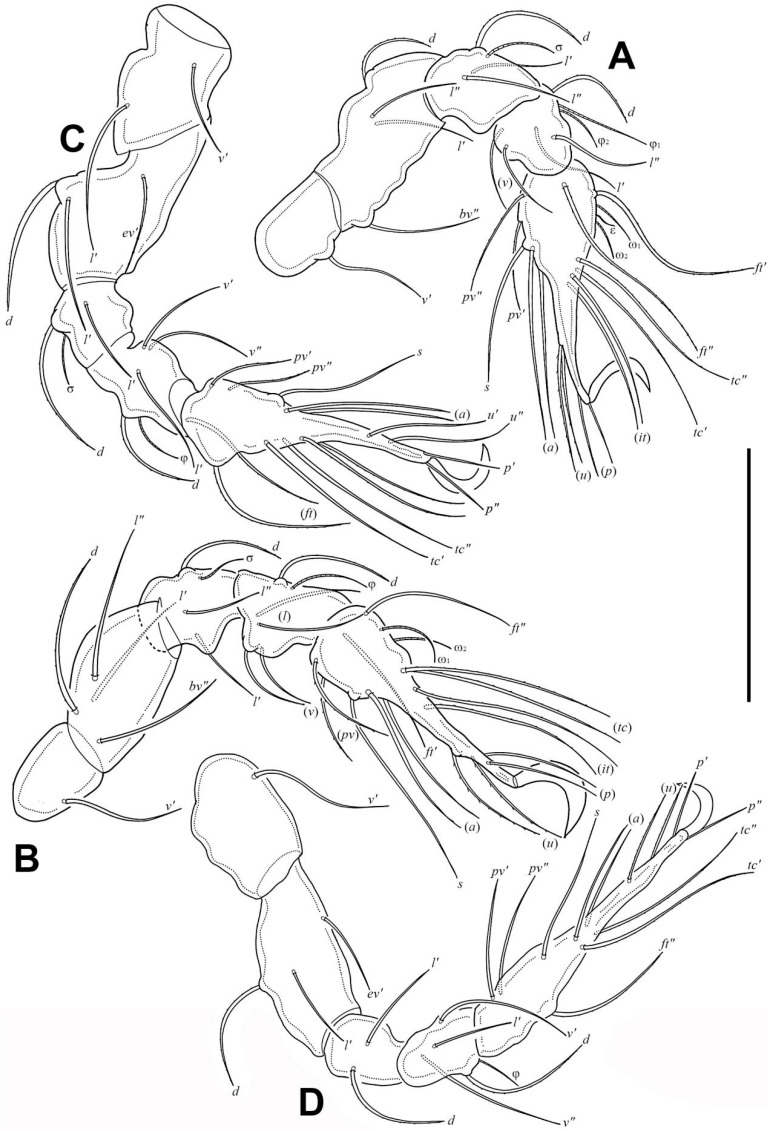
*Eremobelba eharai*, tritonymph, right, antiaxial view, scale bar 50 µm: (**A**) Leg I; (**B**) Leg II; (**C**) Leg III; (**D**) Leg IV.

**Figure 11:**
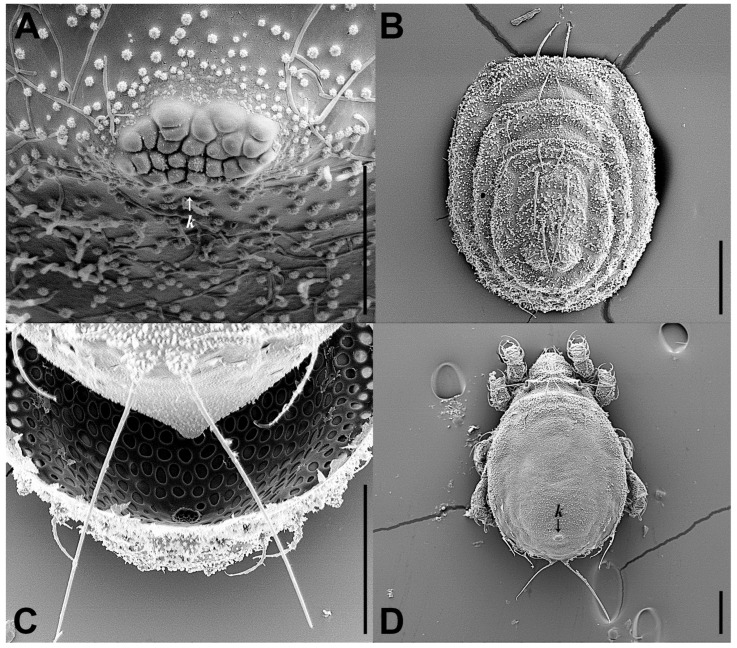
*Eremobelba eharai*, tritonymph, SEM micrographs: (**A**) cornicle, ventral view; (**B**) exuvial scalps, dorsal view; (**C**) notogaster, caudal view; (**D**) dorsal view. Scale bars: (**A**) = 20 µm; (**B**–**D**) = 100 µm.

Nymphs (Figure 2C–E, Figure 6C, Figure 7B, Figure 9, Figure 10 and Figure 11; Table 1). Prodorsal seta *in* short, setae *ro*, *le*, and *ex* middle; setae *ro* and *le* slightly barbed unilaterally in tritonymph, seta *bs* long and barbed bilaterally in nymphs, other prodorsal setae smooth in nymphs. Gastronotum of protonymph with 12 pairs of setae because setae of *p*-series appearing and remaining in deutonymph and tritonymph, setae of *d*-series lost and remaining absent in all nymphs. Gastronotal setae *h*_1_ and *h*_3_ long, *c*_1_ middle, other setae short in nymphs; setae *h*_1_ and *h*_3_ slightly barbed bilaterally, *h*_2_ slightly barbed unilaterally, other setae smooth in protonymph; setae *h*-series slightly barbed unilaterally and other setae smooth in deutonymph and tritonymph; seta *c*_3_ vestigial, with only alveolus visible in nymphs. All setae of protonymph, and gastronotal setae of deutonymph and tritonymph inserted on small apophyses.

Genital valves of protonymph with a pair of setae, and two pairs added in each deutonymph and tritonymph, all short and smooth. Anal valves of protonymph with three pairs of setae, deutonymph and tritonymph with two pairs each, all short and smooth. Aggenital valves of protonymph without setae, deutonymph with two pairs and tritonymph with five pairs of setae, all short and smooth. Adanal valves of protonymph without setae, deutonymph with three pairs and tritonymph with five pairs of setae, all short and smooth. The setae on genital and aggenital valves subequal in length. These setae shorter than those on anal and adanal valves, which also subequal in length to each other.

In all nymphs, cupules *ia* and *im* not observed in granular cerotegument, cupules *ip*, *ips*, and *ih* present, and cupule *iad* appearing in deutonymph and tritonymph; *ip* between setae *p*_1_ and *p*_2_ in nymphs. In protonymph, cupule *ips* lateral–anterior part of anal valves, *ih* lateral–posterior part of *ips*, *ips* and *ih* anterior to seta *p*_3_; *gla* lateral–posterior part of *ih*. In deutonymph, cupule *iad* lateral–anterior part of anal valves, *ips* lateral–posterior part of *iad*, *ih* lateral to *ips*, *gla* lateral to *ih*, and *ips*, *ih*, *gla* anterior to seta *p*_3_. In tritonymph, *iad*, *ips*, and *gla* location same as deutonymph, *ih* posterior to *gla*. Formulae of leg setation and solenidia in tritonymph: I (1–4–3–5–16) [1,2], II (1–4–3–5–15) [1,2], III (2–3–2–4–15) [0,1], IV (1–3–2–4–12) [0–1–0]. Legs monodactylous. Seta *d* present on all genua and tibiae.

Nymphs of *E. eharai* bear the exuviae of previous instars on a specialized dorsal structure termed the cornicle (*k*). This cornicle is characterized by a highly structured surface, composed of numerous distinct, interlocking, and often polygonal small elevations that collectively form a convoluted, papillate texture. This intricate arrangement likely contributes to the mechanical stability required for retaining the successive layers of shed cuticles.

#### 3.1.4. Summary of Ontogenetic Transformations

We summarize ontogenetic transformations of juveniles and adult of *Eremobelba eharai* (Table 2). Except for seta *ex*, all prodorsal setae undergo transformation from juveniles to adult stage. Several traits vary from the larval to the adult stage, such as the numbers and development of notogastral/gastronotal setae (including *c*_3_), epimeral setation, and the setation of the ventral plates.

#### 3.1.5. Comparison of Morphological Ontogeny of *Eremobelba eharai* with *E. geographica* and *E. gracilior*

We compared morphological ontogeny of *E. eharai* studied herein with that of *E. geographica* and *E. gracilior* (Table 3). The adults of these species differ from one another in rostrum shape, the length and shape of notogastral setae, shape of subcapitular seta *h*, presence or absence of *amr*, and shape of epimeral and neotrichous setae. Larvae of these species differ from one another in the shape of seta *bs* and notogastral seta *c*_1_, and in the number of anal valve setae. Nymphs of these species differ from one another mainly in the number of anogenital region setae and in the length of gastronotal seta *c*_3_. In all species, the nymphs carry exuvial scalps of previous instars, which are attached to the gastronotum via cornicles.

Notably, Ermilov [24] recorded adult specimens of *E. gracilior* as possessing 11 pairs of notogastral setae and six pairs of genital setae, whereas Hartenstein [21] reported ten pairs of notogastral setae and five pairs of genital setae. Ermilov [24] conducted a detailed redescription of this species based on topotype specimens received from Professor Roy A. Norton’s personal collection. Therefore, the adult morphological data for *E. gracilior* in this study follow Ermilov [24]. Regarding the morphological ontogeny of *E. gracilior*, only Hartenstein [21] has published relevant records; consequently, juvenile-stage data in this work are derived from Hartenstein [21].

### 3.2. Developmental Stages and Behavior of Eremobelba eharai

#### 3.2.1. Developmental Periods

The life cycle of *E. eharai* includes six stages: egg, larva, protonymph, deutonymph, tritonymph, and adult (Figure 2), and requires four molts to reach the adult stage. *E. eharai* can complete its growth, development, and reproduction with active dry yeast as its sole food source.

During the developmental tracking, 15 individuals of *E. eharai* died during development, and 16 individuals failed to lay eggs within 60 days after reaching adulthood. These individuals were excluded from the data analysis, resulting in a total sample size of 91 individuals (Table 4). The eggs hatched into the larvae after 6.47 days, the entire immature life cycle lasted 47.99 days, the adult stage of *E. eharai* required 22.58 days to reach oviposition, resulting in a generation time of 70.57 days.

#### 3.2.2. Dark-Preference and Oviposition Behavior

During the rearing process, several notable behavioral patterns were informally observed in *E. eharai*. The mites exhibited a strong preference for dark environments, consistently moving towards darker areas within the rearing containers and ceasing feeding activity when exposed to light. Under individual rearing conditions, this species was able to successfully complete the full egg-to-egg developmental cycle, providing preliminary evidence of parthenogenesis. Furthermore, *E. eharai* shows a preference for aggregating during oviposition, often laying eggs on active dry yeast granules. A unique locomotory behavior was observed, wherein individuals shook their first pair of legs three to four times per step, similar to *E. gracilior* [21]. Throughout the study period, no instances of cannibalism were observed, either among living conspecifics or towards dead mites, regardless of food availability.

## 4. Discussion

### 4.1. Morphological Variations of E. eharai Studied Herein Compared with Original Description

Our specimens from Beijing generally align well with the diagnostic characters of *Eueremaeus eharai* as originally described from Liaoning Province [27]. However, several morphological discrepancies were noted, including variation in the presence and shape of certain setae and differences in chaetotactic formulas. We interpret these discrepancies through the following perspectives.

Intraspecific variation: The observed differences likely fall within the range of intraspecific morphological plasticity. In oribatid mites, geographically separated populations may exhibit subtle morphological variations due to environmental factors or genetic drift [28,29,30,31,32], such as the transition of prodorsal setae from smooth to barbed. However, core morphological characters, prodorsal setae *le* inserted on a pair of separated ridges lacking a connecting transverse ridge, stellate subcapitular seta *h*, and several epimeral setae, particularly the presence of eight pairs of stellate neotrichous setae in the aggenitoadanal region, remain highly conserved.

Methodological and Observational Differences: The discrepancies between the original description and our findings are primarily attributable to methodological differences. The original description was based on observations under a compound microscope [27], whereas our study also incorporated SEM. Under SEM, we discovered several new features: the prodorsal setae *le*, *ro*, *in*, and *bs* are barbed (Figure 1C,D); the presence of *amr* and seta *ex* (Figure 1A,D); the detection of additional *lt* and epimeral seta *1c* which may have been concealed or insufficiently resolved by compound microscope.

To sum up, definitive resolution of whether the Beijing population represents intraspecific variation or a distinct species requires the integration of additional diagnostic morphological characters with consistent genetic divergence [31,32]. Accordingly, we conservatively assign the Beijing population to *E. eharai*.

### 4.2. Development and Behavior of Eremobelba eharai

#### 4.2.1. Life Cycle Duration and Behavioral Ecology

Compared to congeners *E. gracilior* and *E. geographica* [18,21], *E. eharai* exhibited a notably faster developmental rate (Table 5). Although environmental factors influence the life-history cycle of oribatid mites [33,34,35,36,37,38,39], there are inherent differences in the developmental cycles of different species [15,40]. Furthermore, the integration of behavioral observations provides an ecological dimension to the life cycle data. The observed negative phototaxis of *E. eharai* may be associated with a reduced risk of desiccation [37,41,42]. Their tendency to oviposit gregariously directly onto the food source may be related to offspring food acquisition [14].

#### 4.2.2. Preliminary Evidence for Parthenogenesis and Unresolved Questions

As the first investigation of reproduction in *E. eharai*, our study provides preliminary evidence for parthenogenesis, thereby revealing potential diversity in reproductive modes within the genus *Eremobelba*, in which *E. geographica* reproduces sexually [18]. However, consistent with the rigorous validation criteria for oribatid parthenogenesis [43,44], our study has limitations that warrant acknowledgment.

First, our isolation rearing spanned only a single generation. This is a limitation because verifying the stability of a reproductive trait across multiple generations is essential to rule out sporadic asexual development or the influence of cryptic genetic factors [45,46]. Second, we did not conduct cytological or molecular analyses [44,47,48]. Additionally, the 16 non-ovipositing adults could reflect delayed reproduction, which is common in temperate oribatid mites with long life cycles [15,49]. An alternative possibility is the non-functional spanandric males [50,51,52]. Distinguishing between these hypotheses would require longer post-maturation monitoring.

Despite these limitations, as emphasized by Norton et al. [46], parthenogenesis in understudied groups of oribatid is often overlooked, and our data provide an initial foundation for further research on this species’ reproductive biology.

## Figures and Tables

**Table 1:** Measurements (Mean ± SD) of some morphological characters of juvenile stages and adult of *Eremobelba eharai* (*n* = 10).

Morphological Characters	Larva	Protonymph	Deutonymph	Tritonymph	Adult
Body length	255 ± 3.99	331 ± 4.95	417 ± 17.82	546 ± 26.44	632 ± 27.50
Body width	162 ± 4.16	197 ± 5.49	251 ± 7.78	340 ± 5.87	362 ± 7.08
Prodorsum length	80 ± 3.68	105 ± 3.07	118 ± 2.64	180 ± 3.33	227 ± 13.35
Length of seta *ro*	27 ± 1.32	34 ± 1.35	43 ± 1.26	49 ± 2.17	58 ± 4.19
seta *le*	29 ± 2.30	35 ± 1.78	44 ± 1.64	50 ± 1.90	65 ± 7.54
seta *in*	5 ± 0.32	8 ± 0.53	13 ± 0.82	14 ± 0.88	48 ± 2.90
seta *ex*	26 ± 1.27	32 ± 2.86	34 ± 2.12	47 ± 3.92	55 ± 6.48
seta *bs*	125 ± 6.79	139 ± 10.31	165 ± 7.17	186 ± 14.98	164 ± 17.54
seta *c*_1_	56 ± 3.43	70 ± 2.81	80 ± 3.98	110 ± 15.17	39 ± 3.78
seta *c*_2_	43 ± 2.31	36 ± 2.12	43.2 ± 2.74	60 ± 11.20	56 ± 4.49
seta *c*_3_	107 ± 4.48	0	0	0	lost
seta *da*	84 ± 4.40	lost	lost	lost	lost
seta *dp*	180 ± 8.38	lost	lost	lost	lost
seta *la*	130 ± 4.90	33 ± 1.93	43 ± 2.00	49 ± 5.95	113 ± 10.88
seta *lp*	77 ± 3.45	22 ± 1.20	23 ± 1.78	33 ± 5.06	128 ± 7.99
seta *h*_1_	149 ± 7.25	182 ± 7.77	242 ± 20.34	280 ± 21.96	115 ± 14.24
seta *h*_2_	25 ± 3.07	40 ± 2.32	44 ± 2.21	73 ± 8.47	130 ± 7.70
seta *h*_3_	12 ± 1.90	168 ± 11.29	215 ± 18.78	265 ± 18.98	121 ± 11.72
seta *p*_1_	nd	23 ± 1.40	37 ± 1.14	57 ± 9.81	93 ± 17.68
seta *p*_2_	nd	22 ± 1.51	32 ± 1.37	36 ± 3.22	118 ± 13.56
seta *p*_3_	nd	20 ± 0.99	27 ± 1.43	30 ± 4.19	122 ± 15.58
genital opening	nd	29 ± 2.44	51 ± 4.00	74 ± 9.57	83 ± 6.34
anal opening	58 ± 2.00	75 ± 1.89	80 ± 2.26	113 ± 11.12	117 ± 8.59

Note: nd—not developed.

**Table 2:** Ontogenetic transformations of juveniles and adult of *Eremobelba eharai*.

Morphological Characters	Larva	Protonymph	Deutonymph	Tritonymph	Adult
Shape of seta *ro*	smooth	smooth	smooth	barbed unilaterally	barbed unilaterally
seta *le*	smooth	smooth	smooth	barbed unilaterally	barbed unilaterally
seta *in*	smooth	smooth	smooth	smooth	barbed bilaterally
seta *ex*	smooth	smooth	smooth	smooth	smooth
seta *bs*	barbed bilaterally	barbed bilaterally	barbed bilaterally	barbed bilaterally	barbed unilaterally
Notogastral/Gastronotal setae (pairs)	12	12	12	12	11
Notogastral/Gastronotal seta *c*_3_	present	vestigial	vestigial	vestigial	lost
Formula of epimeral setae	3–1–2	3–1–3–1	3–1–3–2	3–1–3–3	3–1–3–3
Genital (valves) setae (pairs)	nd	1	3	5	6
Aggenital (valves) setae (pairs)	nd	nd	2	5	neotrichous (17–19 pairs total)
Anal (valves) setae (pairs)	1	3	2	2	2
Adanal (valves) setae (pairs)	nd	nd	3	5	neotrichous (17–19 pairs total)

Note: nd—not developed.

**Table 3:** Compared morphological ontogeny of *E. eharai* studied herein with that of *E. geographica* and *E. gracilior*.

Morphological Characters	*E. eharai*	*E. geographica*	*E. gracilior*
**Adult**			
Rostrum shape	rounded	rounded	triangular
Notogastral setae shape	very curved ^1^, smooth	slightly curved, smooth	slightly curved, barbed
Notogastral seta *c*_1_ length	shorter than *c*_2_	as long as *c*_2_	as long as *c*_2_
Subcapitular seta *h* shape	stellate branches	ciliate branches	ciliate branches
*amr*	present	absent	absent
Epimeral setae *1b, 3b, 3c, 4a* shape	stellate branches	setiform	ciliate branches
Epimeral setae *4b*	setiform	setiform	phylliform
*3c* and *4c* inserted on tubercles	yes	no	yes
Stellate neotrichous setae	yes	no	no
**Tritonymph**			
Gastronotal seta *c*_3_ length	0	145	
Number of genital valve setae (pairs)	5	5	3
Number of adanal valve setae (pairs)	5	4	4
**Deutonymph**			
Gastronotal seta *c*_3_ length	0	115	
Number of genital valve setae (pairs)	3	3	2
**Protonymph**			
Gastronotal seta *c*_3_ length	0	109	
Number of anal valve setae (pairs)	3	3	2
**Larva**			
Seta *bs*	barbed bilaterally	barbed unilaterally	smooth
Gastronotal seta *c*_1_	smooth	barbed unilaterally	barbed
Number of anal valve setae (pairs)	1	2	

^1^ With slender tips curved one to three times. Note: The data for *E. gracilior* are derived from Hartenstein [21] and Ermilov [24], and the data for *E. geographica* are from Seniczak et al. [22]. Blank cells in table indicate that corresponding morphological characters were neither described in original text nor illustrated by line drawings. The numeral “0” in the table indicates that gastronotal seta *c*_3_ is recorded as having zero length because it is vestigial and represented only by an alveolus.

**Table 4:** Duration in days (Mean ± SE and 95% CIs) of various life stages of *Eremobelba eharai* reared on active dry yeast under laboratory conditions (25 ± 3 °C and 80 ± 5% RH).

Developmental Stages	Number of Specimens	Mean (±SE)	95% CIs (Lower–Upper)
Egg Duration	91	6.47 ± 0.05	6.37–6.58
Larva Duration	91	12.15 ± 0.14	11.89–12.42
Protonymph Duration	91	8.66 ± 0.09	8.48–8.84
Deutonymph Duration	91	9.05 ± 0.08	8.89–9.22
Tritonymph Duration	91	11.65 ± 0.14	11.37–11.92
Larva Quiescent Duration	91	17.89 ± 0.17	17.56–18.22
Protonymph Quiescent Duration	91	14.29 ± 0.12	14.05–14.52
Deutonymph Quiescent Duration	91	15.69 ± 0.10	15.50–15.89
Tritonymph Quiescent Duration	91	5.01 ± 0.10	4.81–5.21
Total Immature Duration	91	47.99 ± 0.19	47.62–48.36
Adult–Egg	91	22.58 ± 0.16	22.27–22.89
Egg–Egg	91	70.57 ± 0.16	70.27–70.88

**Table 5:** Experimental conditions and total immature duration of *E. eharai* studied herein with that of *E. geographica* and *E. gracilior*.

Species	Food Sources	Temperature (℃)	Humidity (%)	Total Immature Duration
*E. eharai*	active dry yeast	25 ± 3	80 ± 5	47.99
*E. geographica*	*Aspergillus flavus*	25	100	56–74
*E. gracilior*	*Trichoderma koningii*	20	70–80	68–75

## Data Availability

The original contributions presented in this study are included in the article. Further inquiries can be directed to the corresponding author.

## Abbreviations

Prodorsum: *ro*, *le*, *in*, *bs*, *ex* = rostral, lamellar, interlamellar, bothridial, and exobothridial setae, respectively; *cos* = costula; *abr* = anterobothridial ridge; *mt* = mediobasal tubercle. Notogaster or gastronotum: *c*_1–3_, *da*, *dm*, *dp*, *d*-, *la*, *lm*, *lp*, *h*_1–3_, *h*-, *p*_1–3_, *p*-series = setae; *cr* = crista; *k* = cornicle; *ia*, *im*, *ih*, *ips*, *ip =* lyrifissures or cupules; *gla* = opisthonotal gland opening. Coxisternum and lateral podosoma: *1a*, *1b*, *1c*, *2a*, *3a*, *3b*, *3c*, *4a*, *4b*, *4c* = epimeral setae; *Pd I*, *Pd II* = pedotecta I, II, respectively; *lt* = lateral tubercle; *Va*, *Vm*, *Vp* = anterior, middle, and posterior ventrosejugal tubercles, respectively; *dis* = discidium. Anogenital region: *g*_1_, *g*_6_, *an*_1_, *an*_2_ = genital and anal setae, respectively; *iad* = adanal lyrifissure or cupule. Gnathosoma: *a*, *m*, *h* = subcapitular setae; *amr* = ridge in anterior part of mentum; *cha*, *chb* = cheliceral setae; *Tg* = Trägårdh organ; *sup*, *inf*, *d*, *l*, *cm*, *acm*, *lt*, *ul*, *sul*, *vt* = palp setae; ω = palp solenidion. Legs: σ, φ, ω = leg solenidia; ɛ = famulus; *bv*, *ev*, *d*, *l*, *v*, *ft*, *pl*, *tc*, *it*, *p*, *u*, *a*, *s*, *pv* = leg setae.
